# Climate change and health equity: A public health perspective on climate justice

**DOI:** 10.25646/11772

**Published:** 2023-11-29

**Authors:** Gabriele Bolte, Lisa Dandolo, Sophie Gepp, Claudia Hornberg, Susanne Lopez Lumbi

**Affiliations:** 1 University of Bremen, Institute of Public Health and Nursing Research, Department of Social Epidemiology, Bremen, Germany; 2 Centre for Planetary Health Policy, Berlin, Germany; 3 Bielefeld University, Medical School OWL, Sustainable Environmental Health Sciences, Bielefeld, Germany

**Keywords:** ENVIRONMENTAL JUSTICE, HEALTH EQUITY, HEALTH EQUITY IN ALL POLICIES

## Abstract

**Background:**

The discourse on climate justice has developed from the theoretical approaches and discussions on environmental justice. A central tenet of the concept of environmental and climate justice is that environmental and climate issues cannot be seen in isolation from issues of social justice.

**Methods:**

A conceptual model was developed on the relationship between climate change impacts, social dimensions, adaptive capacities, biological sensitivity, and health equity in order to systematically analyse climate justice. Based on an exploratory literature review and the evaluation of the individual contributions of the status report on climate change and health, the evidence in Germany on social inequalities in exposure to climate change impacts and vulnerability to their direct and indirect health effects was summarised.

**Results:**

This paper provides an overview of the international debate and examples of evidence on climate justice in Germany. Climate justice in the sense of avoidable, unjust social inequalities in exposure, vulnerability, and the effects of climate mitigation and adaptation measures on health inequalities is still insufficiently addressed in Germany.

**Conclusions:**

A consistent integration of equity issues into climate policy is necessary. With reference to the international literature, options for action and research needs are identified.

## 1. Introduction

Social inequalities in health opportunities and disease risks are one of the greatest challenges for public health. It is a central goal to reduce or avoid these inequalities, which are assessed as unjust and preventable [1]. The greatest potential is attributed to those public health measures that address the underlying socioeconomic and environmental living conditions [2].

The broad spectrum of health impacts of climate change has been widely described [3–7]. It is now recognised that climate change impacts can have long-term, amplifying effects on social inequalities and especially on poverty [8]. There is a need for further research, especially with regard to structural consequences (e.g. economic losses, political destabilisation of states or regions with effects on the individual socioeconomic situation), destruction of infrastructure, and simultaneously increased demands on the capacities of healthcare and long-term consequences, especially for psychosocial health [3, 9]. Early signs of social tipping points in the process of destabilisation of societies through climate change impacts are of particular importance [8].

Considering the findings of environmental justice research, it is to be expected that climate change impacts increase social inequalities in health on a global as well as on a national or regional level [10, 11]. In particular, people living in poverty are more affected by climate change impacts due to higher exposure and sensitivity as well as lower adaptive capacity [11]. At the same time, they contribute much less to greenhouse gas emissions. Public health interventions in the context of climate change should therefore take into account the vulnerability of socially disadvantaged populations and focus on eliminating health inequalities [10].

This paper aims to provide a first overview of the evidence on climate justice in Germany from a public health perspective. Based on a conceptual model, social inequalities in exposure to climate change impacts and in vulnerability (biological sensitivity, adaptive capacity) to the direct and indirect health effects of climate change impacts are considered. Recommendations for public health research and climate justice monitoring are given and options for action for just climate adaptation practices in Germany are identified.

## 2. Conceptual framework: Climate justice from a public health perspective

### 2.1 Climate justice and environmental justice

When considering anthropogenic climate change on a global scale, the concept of climate justice is concerned with the equitable distribution of the burdens caused by climate change in view of the unequal shares of causation, i.e the greenhouse gas emissions of states, especially of the Global North, both in the past and at present. Furthermore, it is concerned with supporting the management of climate change impacts and structural change towards a climate-neutral, just social and economic order. Global climate justice thus includes social justice and the recognition of human rights worldwide [12, 13].

The discourse on climate justice has developed from the concepts and discussions on environmental justice [14–16]. An intersectionality perspective considers the impact of interactions between different dimensions of inequality for the social situation and processes of privilege or disadvantage [17]. For the concept of environmental justice, it is a central tenet that environmental issues cannot be seen in isolation from issues of social justice.

The climate justice debate also includes the three central justice dimensions of the environmental justice discourse [18, 19]: distributive justice, procedural justice, and the recognition of the dignity and rights of all individuals and population groups as well as their cultures, values, and perspectives (i.e. no stigmatisation or discrimination) [12, 20]. In addition, there is the aspect of the polluter pays principle [21] and the aspect of restorative justice in questions of just compensation for the consequences of climate change and the protection of particularly vulnerable population groups [22].

Intergenerational justice refers to unequal distributions of burdens between generations. Intragenerational justice refers to unequal distributions of the impacts of climate change, the costs and burdens of climate mitigation and adaptation measures within a generation [23]. Both inter- and intragenerational justice are about fairness between populations, states, and generations [12].

According to Buse and Patrick [16], climate justice from a public health perspective means recognising the existing imbalance between causation and harm, taking appropriate action to correct this imbalance, and developing solutions for climate mitigation and adaptation. Any actions should be guided by human rights, empowering or increasing the resilience of individuals and communities, and improving health and well-being.

Vulnerability is a key concept with different definitions in many research and policy fields [23, 24]. While exposure to climate change impacts is often considered a component of vulnerability in the climate change discussion [16], in our concept we refer to the definition for individual-level vulnerability in the field of environmental justice research [25, 26], to the definition in the current Sixth Assessment Report of the Intergovernmental Panel on Climate Change (IPCC) [12], and to the definition of social vulnerability of the European Environment Agency [27]. Vulnerability at the individual level includes previous cumulative exposures, pre-existing diseases, malnutrition, lack of resources or knowledge, and other physiological aspects, i.e. adaptive capacity and biological sensitivity. An intersectionality approach is central to understanding differences in vulnerability between social groups [8]. The conceptual distinction between vulnerability and current exposure is important when planning interventions to reduce health inequalities in climate change impacts.

### 2.2 International evidence in the Global North

In addition to the global perspective on climate (in)justice described above, another perspective is the lack of climate justice in the sense of social inequalities in individual exposure, in sensitivity to the health effects of climate change impacts, or in adaptive capacities within states or societies.

An example of this is given for countries in the Global North: in many European countries, socially disadvantaged population groups live in densely populated, urban areas with higher exposures to air pollutants, noise, and heat. For example, in the United Kingdom, it has been shown for London and Manchester that low-income populations are more likely to live in urban heat islands [27]. A study of 175 urban areas in the United States found that the average summer daytime surface temperature is higher in those urban areas where People of Colour or low-income populations live [28]. Populations with a low socioeconomic position often have a higher risk of heat-related morbidity and mortality [27, 29–32].

Social disadvantage can be associated with lower adaptive capacities, e.g. when socially disadvantaged population groups live in poorly insulated dwellings that heat up easily and have no possibility to implement heat protection measures or move to better housing. Another example from the United Kingdom is the lack of insurance cover against flood risks for financial reasons [33].

The aftermath of Hurricane Katrina on the U.S. Gulf Coast in 2005 is a prominent example of how socially disadvantaged, marginalised population groups can be particularly affected in their neighbourhoods, not only directly by flooding, but also by inadequate disaster management and delays in aid [34].

Extreme weather events can impair healthcare functions due to the impact on infrastructures of general interest (e.g. transport, social infrastructure) [35], which in turn particularly affects socially disadvantaged population groups due to higher morbidity and few opportunities to evade.

### 2.3 A conceptual model on climate justice

Understanding the interplay of social differences in exposure, biological sensitivity, and adaptive capacity is crucial for assessing the impacts of climate change on human health in terms of health inequalities and equity.

**Annex Figure 1:**
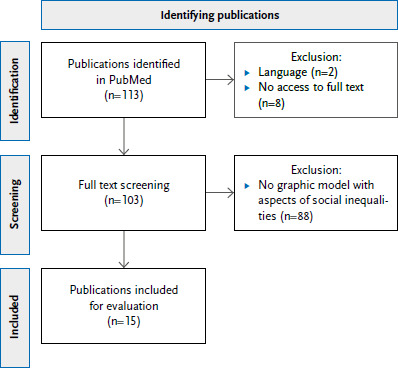
Flow chart for identifying publications with graphical representations of concepts or models on climate justice and health Source: Own representation

As a basis for our own conceptual model development, relevant and current concepts and models on climate justice and health impacts or on the importance of structural and individual dimensions of social inequalities for the health effects of climate change impacts were identified through a literature search. For this purpose, a search was conducted in the MEDLINE database via PubMed on February 07, 2023, using a combination of keywords for the areas climate change, health, social justice, and models/concepts (Annex Table 1). Out of 113 hits, eight were excluded due to lack of full text availability and two due to language. A total of 103 full texts were screened by one author (LD) for relevant graphical models that included aspects of social inequalities and social justice. In a first round, 15 articles with graphical models were included (Annex Figure 1), of which five models were considered particularly relevant after discussion by two authors (GB and LD) [36–40]. Other models (e.g. [41, 42]) were obtained from grey literature, reviews on climate change and health, and references from publications. Furthermore, reference was made to the so-called risk propeller from the Sixth Assessment Report of the IPCC, which refers to the dynamic interactions between climate-related hazards, exposures, and vulnerability [12]. A framework on vulnerability to health effects of climate change impacts [43] published after the literature search did not contribute any new aspects of climate justice.

**Figure 1:**
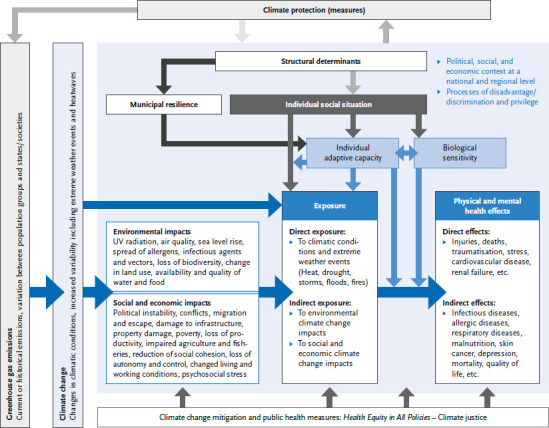
Relationship between climate change impacts, social dimensions at structural and individual level, adaptive capacities, biological sensitivity, and health equity Source: Own representation

Figure 1 shows the model we developed. It visualises the relationship between the direct effects of climate change, environmental, social and economic impacts, social dimensions at structural and individual levels, vulnerability (adaptive capacity, biological sensitivity), and health effects with a focus on climate justice within a spatially limited area (e.g. state or region). The consequences of climate change, i.e. changes in global climatic conditions including extreme weather events and heatwaves, are understood in the model as an overarching hazard (cf. [12, 44]). This affects larger areas, e.g. cities (heatwaves), regions (flood disaster in parts of North Rhine-Westphalia and Rhineland-Palatinate 2021, in Thessaly, Greece 2023) and states (flood disaster in Pakistan 2022). The level of individual exposure is decisive for health effects. It includes direct exposure to climatic conditions and extreme weather events, exposure to environmental climate change impacts, and exposure to social and economic climate change impacts. Individual exposure varies according to time and place and what adaptation measures are taken. During a heatwave, the installation of sun shades is an example of an individual adaptation measure. An example of a collective/municipal adaptation measure is the provision of publicly accessible cool places.

Structural determinants embedded in the political, social, and economic context with the associated processes of disadvantage/discrimination and privilege include, for example, the supply and accessibility of social care and healthcare, and influence an individual’s social situation and resilience at the municipal level. Municipal resilience, for this purpose, follows the definition for urban resilience as the capacity of an urban system and its population to respond resiliently to crises or disasters while adapting and transforming itself for sustainable urban development [45], with an extension to rural areas. Municipal resilience encompasses robustness and adaptability [46].

In our model (Figure 1), the social situation is viewed from an intersectionality perspective. People can be discriminated against in multiple ways along different dimensions of inequality and characteristics, depending on context. For example, people with a history of migration and a physical disability can be particularly disadvantaged in their search for housing. Accordingly, social situation is understood as an interaction of different inequality dimensions [40], which leads to intersectional discrimination, unequal power relations, and unequal access to social and material resources. Differences in the health effects of climate change impacts through effect modification can be caused by social differences in individual adaptive capacity and individual sensitivity. These are in turn influenced by the individual social situation and, at the contextual level, by municipal resilience and structural determinants.

Individual adaptive capacity is constituted by skills, material and social resources, as well as knowledge of how to adapt to changing climate conditions and the associated ecological, social, and economic impacts and to respond to (disaster) events, i.e. both short-term response capacity and long-term adaptive capacity. Individual adaptive capacity also includes coping as a stress management strategy [47] and individual resilience as variable psychological resistance due to personal and social protective factors [48]. Individual adaptive capacity influences the type and extent of individual exposure to given climatic conditions or extreme weather events, ecological, social, and economic climate change impacts in the sense of exposure variation [49, 50]. Furthermore, it can modify the health effects of exposures.

Individual biological sensitivity or susceptibility refers primarily to biological aspects such as altered physiological reactions to exposures due to pre-existing diseases, maturation or ageing processes, or genetic factors. Social inequalities in the material and social living environment influence biological sensitivity via mechanisms such as psychosocial stress and allostatic load as a result of chronic stress reactions or the regulation of gene expression (cf. embodiment concept of ecosocial theory) [51–54]. Physiological conditions due to pre-existing diseases or age can in turn influence the individual adaptive capacity. Individual biological sensitivity acts as an effect modifier, i.e. it modifies the health effects of an individual’s exposure [49, 50].

At the regional and national level, but even more so at the global level, the social and economic impacts of climate change influence the individual’s socioeconomic position and social situation through impoverishment and even up to the loss of livelihoods and compulsion to migrate and seek refuge. In addition, there are impacts on structural determinants, such as societal destabilisation and deterioration of infrastructure. The social and economic impacts of climate change therefore have wider implications for health inequalities, even independently of individual exposure to direct and environmental climate change impacts.

The model refers to the fact that population groups and countries contribute and have contributed differently to greenhouse gas emissions. For example, the average greenhouse gas emissions/year of the wealthiest 10% of households in Germany are approximately six times higher than the emissions of all households with incomes below the median [55]. The type and extent of climate protection measures implemented by a state are influenced by the political, social, and economic context. Climate protection measures can in turn affect social inequalities and have co-benefits for health (e.g. nature conservation areas as CO_2_ sinks as well as recreational and leisure areas, see Section 4 Integrating the equity perspective).

Figure 1 shows schematically that climate adaptation and public health measures can influence the different levels of exposure, adaptive capacity, sensitivity, and health. In the context of structural social inequalities, a Health Equity in All Policies approach [24] is an essential prerequisite for achieving climate justice.

## 3. Evidence in Germany

To determine the evidence in Germany regarding climate justice, i.e. social inequalities in exposure to climate change impacts, vulnerability and health effects, the following literature searches and evaluations were conducted: (1) a systematic literature search of peer-reviewed publications, (2) an exploratory search for grey literature, and (3) an evaluation of all other thematic articles within the status report on climate change and health [56–65]. The authors of these articles, published in the Journal of Health Monitoring in 2023, were additionally asked for further literature references on studies in Germany.

### 3.1 Systematic literature review of studies in Germany

The systematic search of published peer-reviewed studies in Germany was conducted on February 15, 2023 in the MEDLINE database via PubMed. Keywords for the domains social justice, climate change, and Germany were used (Annex Table 2) and the search was limited to title and abstract as well as MeSH terms. There was no limitation in terms of publication year or type. One author (SLL) performed the data retrieval, two authors (GB and SLL) independently performed the screening of title and abstract for the identified publications. All studies were included that examined the significance of social dimensions for exposure to climate change impacts or their health effects in Germany. Discrepancies in the selection of studies were discussed by the whole team and inclusion was decided by consensus.

**Annex Figure 2:**
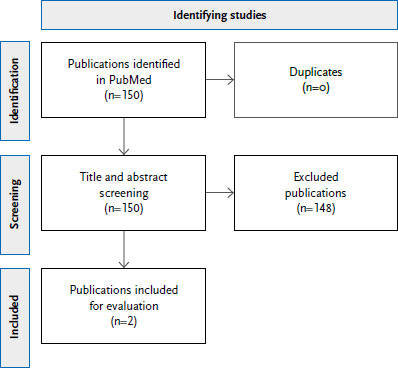
Flow chart for identifying studies on the significance of social dimensions for exposure to climate change impacts or their health effects in Germany Source: Own representation

The systematic search in PubMed yielded 150 hits. After title and abstract screening, two studies were included as suitable for evaluation (Annex Figure 2). Both studies investigated subjective heat stress and were conducted in the cities of Cologne [66] and Dresden [67], respectively.

In both studies, residents of selected urban areas were asked about their perceived heat stress and adaptation strategies. In Cologne, four urban areas were selected based on the criteria of socioeconomic and urban climatic living conditions. Within the urban areas, people aged 65 and over were surveyed [66]. In Dresden, two urban districts were selected on the basis of building and social structural data [67]. In Cologne, 258 persons – 131 women and 127 men – aged 65 to 93 years participated in the survey; in Dresden, 661 persons with an average age of 47 (district A, 45% women) and 48 years (district B, 51% women), respectively.

The Dresden study showed that people from a neighbourhood with a very high social burden (according to the social index of the Dresden Education Report), highly built-up areas, a low proportion of neighbourhood greenery and only one park in the catchment area felt more frequently subjectively burdened by heat in general, heat in the neighbourhood and heat in the home during the day than people from a neighbourhood with a low social burden according to the social index, less built-up areas, a medium to high proportion of neighbourhood greenery and three parks in the catchment area. Furthermore, the first group was less likely to rate their subjective health as good [67]. Heat-related symptoms during summer heat events were mentioned more frequently in the first neighbourhood. There were also differences in the participants’ adaptation strategies: in the neighbourhood with very high social burden, the choice of clothing, avoidance/protection from heat, frequenting of green spaces, frequenting of cool rooms inside the residence, and avoiding the midday sun were mentioned less frequently than in the neighbourhood with low social burden [67].

The Cologne study [66] found no differences in the subjective heat stress of the participants between the two urban areas with high and the two urban areas with low objective heat exposure in a comparison of four selected urban areas. A further analysis in combination with socioeconomic parameters of the urban areas was not reported. Differences were observed at the level of individual social parameters: women, people with lower income, lower school education and people with poorer health status were more likely to feel subjectively burdened by heat. Age differences were not observed in this study population, which only included people aged 65 and older. Subjective heat stress correlated positively with the number of adaptation strategies implemented. Differences in the type of adaptation strategies were reported between different groups – women, for example, were more likely than men to report using thinner bedding during heat events or cooling their arms with water, and people with higher education were more likely to report increasing their fluid intake. Further interpretations of the study results are not possible as the baseline prevalences for the individual groups were not reported and the statistical analysis did not account for multiple testing.

### 3.2 Further evidence from exploratory literature search for Germany

The exploratory search of grey literature and references from publications yielded only isolated information on the relevance of social dimensions for exposure to climate change impacts, for their health effects, or for adaptation capacities in Germany. This information is briefly presented below.

Darabi et al. [68] used data from the Berlin Environmental Justice Atlas from 2009 to 2011 to investigate whether environmental stressors including heat mediate the effect of poverty on mental health. Individual-level data on sociodemographic characteristics and health were obtained from 478 individuals, 244 women and 234 men aged between 18 and 68 years, from eleven planning areas in Berlin-Mitte via interviews in 2011. At the level of the eleven areas studied, thermal stress (physiologically equivalent temperature) did not correlate with contextual poverty (recorded as the proportion of the residential population receiving long-term unemployment benefit), in contrast to air pollution and the availability of public green spaces. The publication did not provide information on the variation of thermal burden between the eleven studied areas. The environmental factors studied were not associated with mental health.

The 2019 Environmental Justice Report for Berlin describes that 228 out of 447 areas were affected by high bioclimatic stress. 65 of 228 areas with high bioclimatic stress also had a disadvantaged social structure. Data on the status and dynamics of the four indicators unemployment, long-term unemployment, transfer payments (Social Code (SGB) II and XII), and child poverty (transfer payments SGB II for under 15-year-olds) were included in the overall social inequality index. Areas with a disadvantaged social structure, in which cumulative social problem situations could be observed, were designated as areas in need of special attention [69]. There were 40 areas with this double burden in the extended inner city area. In the updated report from 2022, only the integrated multiple burden of environment and social disadvantage was presented; information on social differences in bioclimatic stress alone is not provided in an overview of all areas [70].

A spatial analysis of data from the German National Association of Statutory Health Insurance Physicians on state-insured patients from 2009 to 2015 showed a positive association in the multivariable analysis between the proportion of employees with an academic degree and household income at the district level with the prevalence of malignant melanoma of the skin (International Classification of Disease ICD-10: C43), adjusted for average regional sunshine duration [71]. Household income at the district level was associated with the prevalence of basal cell carcinoma and squamous cell carcinoma (ICD-10: C44), adjusted for regional average UV radiation burden. The authors suspect the cause to be higher travel activity of persons with higher incomes in the past decades [71]. When interpreting these data, however, it must be taken into account that (1) the data show prevalences of diagnosed and treated cases of the disease and not prevalences among the entire resident population in Germany and that (2) socioeconomic data aggregated at the district level were used for the year 2014.

Bubeck and Thieken [72] investigated the subjective recovery of affected persons 18 months after the 2013 Elbe and Danube floods. For this purpose, they conducted telephone interviews with 710 households. The importance of event characteristics (e.g. flood depth and duration), circumstances of the recovery process (e.g. condition of buildings, duration of compensation payments), socioeconomic factors (e.g. age, gender, education, income, insurance coverage, home ownership) and psychological factors (e.g. mental preoccupation with the flood, stress resistance, perceived safety from future events, trust in others) were investigated. Analysis of socioeconomic characteristics revealed lower subjective recovery among women compared to men, those with poorer health or disability, and those with home ownership. Overall, socioeconomic characteristics and psychological factors were more important for long-term subjective recovery than event characteristics or circumstances of the recovery process. Based on their results, Bubeck and Thieken [72] conclude that reconstruction processes after flood events should not be focused exclusively on particularly damaged areas, but should take into account socioeconomic characteristics of the affected population. Recovery measures should especially support socially vulnerable groups, i.e. people with disabilities, poor health, and low financial resources [72].

Based on data of the German Socioeconomic Panel for over 10,000 households in Germany in the period 2012 to 2020, Osberghaus and Abeling [73] investigated whether there are differences in exposure or vulnerability to heat between households with relative income poverty and households without relative income poverty. There were no social differences in the extent of heat exposure at the place of residence (recorded as mean daily minimum ambient temperature in summer at the place of residence) and in exposure parameters for urban heat islands (population density, living on the top floor of a multistory apartment building). However, it must be borne in mind that no distinction was made between urban and rural regions in the analysis. The authors were able to show differences in vulnerability parameters (age, gender, household size, health status) as well as adaptive capacity (heat protection measures already taken in the home, potential for future implementation, e.g. self-efficacy, locus of control, expected costs (determined in relation to the installation of air conditioning)) between households with and without relative income poverty. The results of the study support the approach of not exclusively considering exposure differences when assessing climate justice.

In their report on gender aspects of climate policy from an intersectionality perspective, Spitzner et al. [74] point out that particularly single mothers and female pensioners in Germany more often live in housing that is poorly equipped in terms of heat protection. Causes for this could be income poverty, but also discrimination on the housing market.

### 3.3 Evaluation of the articles of the status report on climate change and health

The third step of the analysis involved the evaluation of the articles of the German status report on climate change and health with regard to vulnerable populations and the influence of social determinants on the health impacts of climate change impacts. Two authors (LD and SG) extracted all statements from the articles on climate change impacts on infectious diseases [56–59] and on non-communicable diseases [60–65]. A distinction was made between exposure to climate change impacts (Table 1), sensitivity to the health effects (Table 2) and adaptive capacity (Table 3). Social determinants were defined according to the PROGRESS-Plus Framework [75, 76]. The PROGRESS acronym includes eight dimensions: (1) place of residence, (2) race/ethnicity/culture/language, (3) occupation, (4) gender, (5) religion, (6) education, (7) socioeconomic status, and (8) social capital. The ‘plus’ stands for other determinants that may be associated with social discrimination, marginalisation and exclusion, such as age or the presence of a disability. Pregnant women and people with pre-existing diseases were included in the tables as particularly vulnerable population groups for physiological reasons. In addition, lifestyle factors, e.g. sports activities, were included in the evaluation, which may be associated with the social situation, but are not social determinants in the narrower sense.

In the tables of results (Tables 1–3), only those social dimensions according to PROGRESS-Plus that occurred in the evaluated articles are presented. For example, education was not mentioned as a social indicator in the evaluated articles and is therefore not shown as a separate column in the tables. In the tables, it was also noted whether the source for the extracted statements in the respective articles is based on German evidence (GE), European evidence (EE), or more extensive international evidence (IE). Where the source of evidence is a review article, this was marked with the additional abbreviation ‘-R’. Statements without cited references were included in the tables without an additional note. Decisions on the inclusion or exclusion of certain statements and the assignment to the aspects of exposure, sensitivity, or adaptive capacity were made by all authors as a team. The result of this evaluation was presented to the authors of the articles [56–65] for review. Decisions on additions or changes based on the feedback were again made by the entire team of authors. At this point, it should be explicitly pointed out that this third step of the analysis only includes the evaluation of the information in the articles and no further analysis of references mentioned there. For example, the article by Baldermann et al. [62] on UV radiation refers to the S3 guideline ‘Prevention of skin cancer’, which contains further information on social inequalities with reference to various dimensions such as education, socioeconomic status, age, and gender. Thus, this evaluation offers a first insight, but not a fully comprehensive presentation of the evidence in Germany.

#### Differences in exposure

Above all, an individual’s occupation is associated with exposure to climate change impacts and their health effects, for example when working outdoors (Table 1). Recreational behaviour was also listed frequently as a reason for higher exposure, e.g. to infectious diseases, heat, and UV radiation.

#### Vulnerability – differences in sensitivity

Table 2 shows which social determinants play a role in relation to differences in sensitivity to climate change impacts. In most articles, age (children, older people) and pre-existing diseases as well as pregnancy are mentioned as reasons for increased susceptibility.

#### Vulnerability – differences in adaptive capacity

Social dimensions for which there is evidence of differences in adaptive capacity are listed in Table 3. Socially different adaptive capacities seem to be particularly relevant with regard to the health consequences of heat and extreme weather events or statements were only made in the articles of the status report concerning these topics. Socioeconomic status, social networks, the presence of a disability, as well as age are mentioned as relevant social determinants for both exposures.

One aspect not shown in Tables 1–3 is the psychological processing of climate change impacts. Gebhardt et al. [65] point out that children and adolescents are particularly vulnerable to the development of mental illness through knowledge of climate change impacts (GE). In addition, it is stated that women report stronger fears in relation to climate change than men (GE). According to international evidence, groups of people with pre-existing structural disadvantages and vulnerabilities are particularly affected by the direct and indirect mental health impacts of climate change (IE-R), while social family structures and education levels are considered resilience factors after extreme weather events and are protective against mental stress (IE-R). Resilience factors related to the indirect psychological impacts of climate change, i.e. the burdens caused by knowledge of climate change, are still largely unexplored (IE-R). For factors such as socioeconomic status, ethnicity, migration history, spatial marginalisation, and intersectional discrimination, there are currently no studies from Germany on the relationship between climate change impacts and mental health, according to Gebhardt et al. [65].

Furthermore, aspects of migration/escape were highlighted [59, 61]. Forced (transnational) migration or escape during extreme weather events due to climate change impacts has a significant effect on health, vulnerability, and social situation.

### 3.4 Conclusion on the available evidence in Germany

Evidence on the different aspects of climate justice in Germany with a reference to health appears to be insufficient overall. Differences in biological sensitivity in relation to direct and indirect health effects have been studied more frequently compared to social differences in exposure, adaptive capacity, or opportunities to participate in decision-making processes. Heat stress is a central topic. A view on cumulative exposures (e.g. occupational heat exposure and simultaneous heat exposure in the home) as well as an intersectionality view on inequalities seems to be missing. For analyses of this kind, data at the individual level are needed.

In the global view on climate justice and especially on the situation in LMIC countries (low- and middle-income countries), it is assumed that women are more vulnerable to the health impacts of climate change. This is justified by societal, cultural, and economic conditions, i.e. structural disadvantage and discrimination based on gender [8, 77]. In the studies considered in this overview, differences between women and men are partly described, but a reference to social dimensions of gender and aspects of gender justice is not made.

#### Limitations

It should be critically noted here that a systematic literature search for this article was only conducted in one database for health-related publications. The explorative search additionally revealed individual studies that could not be found via this database. It can be assumed that there are more studies on social differences in exposure and adaptive capacity from Germany that do not evaluate the results from a public health perspective. The studies identified via the various searches were not subjected to a systematic quality assessment in this article; only individual references to problematic issues such as multiple testing are provided.

It should also be borne in mind that the articles of the German status report on climate change and health [56–65] did not systematically compile the evidence on social determinants and social inequalities for their respective topics. In this respect, the insights from these articles, which are summarised in tabular form above, only represent a part of the evidence presumably available in Germany.

## 4. Integrating the equity perspective into climate change mitigation and adaptation measures – international discussion

*‘Adaptation and mitigation measures to address climate change needed to protect human society must also be planned to protect human rights, promote social justice, and avoid creating new problems or exacerbating existing problems for vulnerable populations.’* Levy and Patz [78, P. 310]

According to the Lancet Countdown experts in Europe, the greatest health policy opportunity of this century is to design and implement climate change mitigation and adaptation measures in a way that focuses on health, well-being, and equity [5]. There is a risk that without appropriate climate mitigation and adaptation measures, those negative impacts of climate change that act primarily on the social determinants of health will further increase social inequalities in health [11]. Given the impacts of climate change on livelihoods and social determinants of health, rapid and comprehensive implementation of a climate policy focused on social justice, protection of human rights, and sustainability is crucial [6, 8, 11, 78]. Care must be taken to ensure that climate mitigation and adaptation measures do not contribute to the emergence or widening of existing social inequalities [78–80].

Consistent integration of the equity perspective makes it easier to go beyond incremental adaptation to climate change impacts and increase resilience and sustainability [81]. A climate-resilient healthcare system is characterised by the fact that all people have access to social and health services, vulnerabilities and inequalities are reduced, and a Health in All Policies approach is pursued in cross-sectoral cooperation, e.g. with urban planning [82]. Another example are disaster management strategies for extreme weather events, which should take into account different vulnerabilities of population groups, especially at the local community level [4].

Economic development needs to change, prioritising health-promoting urban development, the use of more efficient and renewable energy sources, and a sustainable and more just food system. Ecological and social determinants of health need to be addressed together to reduce poverty, increase health equity, and enable all people to live within planetary boundaries [83].

Friel [84] proposes the concept of ‘Planetary Health Equity’. It contains the following elements:

1. Embedding policy norms of social equity, environmental sustainability, and well-being
2. Application of these policy norms and implementation in cross-sectoral policies
3. Implementation of a guiding national strategy on climate, equity, and health
4. Resetting the governance of planetary health equity to ensure that there are no vested interests and that there is civil society participation

Internationally, the need for community-based, localised approaches to climate adaptation is emphasised. Climate justice principles can be integrated into public health strategies and interventions for climate adaptation at the community level to increase the resilience of marginalised populations to climate change impacts and other stressors [16, 17]. Interactions between exposures, biological sensitivity, adaptive capacity, and the social determinants of health should be considered location-based from an intersectionality perspective to better understand differences in the health effects of climate change impacts as well as climate adaptation measures and to develop differentiated interventions in a participatory manner with population groups [16, 17, 74].

Urban greening is an essential component of municipal climate adaptation strategies and sustainable, climate-just urban development. Urban greening not only improves urban climatic parameters, but also the exposure to air pollutants and noise. Public green spaces also have health-promoting potential in terms of social interactions in public spaces and physical activity [85]. There is extensive evidence that the availability of and access to urban green spaces is socially unevenly distributed [86, 87]. Urban greening measures can reduce these social inequalities but can also have unintended negative effects: there is a risk of gentrification, i.e. the displacement of poor and socially disadvantaged population groups from neighbourhoods enhanced by urban greening [88, 89]. First analyses in 28 cities in North America and Europe show that there can be temporal correlations between urban greening and gentrification [90]. It is therefore crucial to assess in a methodically sound way the influence of such interventions on the extent of (new) climate and environmental injustices and to include suitable instruments for counteracting them in the planning phase [24].

The Sixth Assessment Report of the IPCC highlights the issues of social justice, different forms of knowledge, e.g. in the local population and in science, the role of power and participation in the implementation processes of climate adaptation measures [8, 12]. When planning adaptation measures, their expected effects on justice issues should be assessed, and the implementation should be accompanied by justice-related monitoring and evaluation. Examples of implementation can be found in an analysis of the climate action plans of the 100 largest cities in the United States [91]: equity aspects have been increasingly taken into account in cities in recent years, especially for the energy, land use, and transport sectors. Measures anchored in the climate action plans to achieve more climate justice were, for example, cooperation with local actors and vulnerable population groups, the establishment of an advisory board for justice issues, the development of instruments to record equity aspects in the planning, implementation, and evaluation of measures, as well as justice indicators to quantify the effects of the measures on justice.

## 5. Recommendations for action and need for research

The public health perspective on climate change and health equity adopted in this article demonstrates the need to fundamentally anchor justice issues in Germany’s national and international climate policies.

The German Strategy for Adaptation to Climate Change (DAS), adopted in 2008, pursues the long-term goal of reducing the vulnerability of natural, social, and economic systems and maintaining and increasing their adaptive capacity. The DAS ties in with the goal of the sustainable development strategy for Germany to harmonise the sustainability dimensions of economy, ecology, and social issues [92]. In the health cluster, the Second Progress Report on the DAS cites as measures, among others, ‘developing outreach to particularly vulnerable groups of the population (e.g. the elderly, people with pre-exisiting conditions, children)’ [93, P. 58] and a better integration of health and environmental monitoring. In the Climate Impact and Risk Assessment 2021 for Germany, the subreport on risks and adaptation in the economic and health clusters [94] mentions measures from the adaptation action plan [93] in the field of human health, such as effectiveness analyses of health-related adaptation measures within the framework of heat-health action plans (HHAPs), establishment of an integrated health and environmental monitoring system, evaluation of the implementation and effectiveness of recommendations and prevention measures, and information materials tailored to vulnerable target groups. Vulnerability is predominantly understood in this context as biological sensitivity. In the Climate Impact and Risk Assessment 2021 for Germany, sub-report on integrated evaluation of climate risks, action requirements and research needs [95], a need for research was identified for the field of human health, regarding socio-spatially differentiated analyses of heat stress and small-scale analysis of heat-related excess mortality. This lack of knowledge on socioeconomic risk constellations in Germany with regard to heat and health was also highlighted in the current analysis of HHAPs and adaptation measures to extreme heat events in Germany [96]. Monitoring of the exposure situation with regard to multiple social and environmental burdens is essential for an adequate assessment by decision-makers. Climate change-compatible urban development requires the integration of aspects of demographic change and environmental justice [97].

In the DAS, the dimensions of distributive, procedural, and recognition justice outlined in this article have not yet been explicitly addressed. Currently, however, equivalent living conditions and equity in the availability of and access to ecological resources as well as in climate change burdens are increasingly being considered with reference to the concept of environmental justice. In the German federal government’s draft law for a Climate Adaptation Act (KAnG), the following overarching goals are mentioned: ‘The resilience of ecological systems and society to climate change, which will continue to progress in the future, is to be increased in order to preserve equivalent living conditions and contributions are to be made to national and international efforts in climate adaptation. The increase in social inequalities due to the negative effects of climate change is to be prevented.’ (§1 of the KAnG [98], original language German)

The state of knowledge and conceptual considerations on climate justice from a public health perspective result in the recommendations for action and research needs for Germany presented in Table 4.

## 6. Conclusions

Climate justice in the sense of avoiding or at least reducing social inequities in exposure to climate change impacts, in vulnerability related to the direct and indirect health effects of climate change impacts, and in the effects of climate mitigation and adaptation measures on health inequities is still insufficiently addressed in Germany. Systematic studies as well as the consistent integration of social justice and health equity into climate policy are largely lacking thus far. Overarching recommendations for action for Germany were derived in this article from the international discourse and from examples of the integration of justice aspects into the planning, implementation, and evaluation of climate measures from an intersectionality perspective. Even though specific data and evidence for Germany are often still missing, it is possible to start acting in line with the precautionary principle, based on knowledge about environmental justice and health equity, for a transformation to resilient, climate-just, sustainable, and health-promoting living environments for all.

**Info box 1 Heat-health action plans as an instrument for climate justice**

Heat-health action plans (HHAPs) are a key municipal instrument for protecting human health from heat. In 2020, the conference of German federal health ministers declared a requirement of HHAPs at the municipal level in Germany within five years. One basis for this are the recommendations for the development of HHAPs for the protection of human health developed by a working group comprised of federal and state actors and published in 2017 by the Federal Ministry for the Environment, Nature Conservation, Building and Nuclear Safety [108]. The short-, medium-, and long-term measures of municipal HHAPs should take into account the specific local conditions. So far, the view of biological heat sensitivity has predominated in recommendations and working aids for municipal HHAPs. Socially disadvantaged population groups are not in focus, with the exception of homeless people as a group particularly affected by heat and simultaneously vulnerable.

If future HHAPs were designed from the outset to address social inequalities in both heat exposure and vulnerability with regard to health consequences from an intersectionality perspective, and the evaluation of HHAPs was carried out systematically with regard to inequality effects, this could be an important step towards more climate justice in Germany.

Structural heat prevention measures, especially in cities, require interdisciplinary and intersectoral cooperation. The necessity of integrating the different sectors, especially health, environment, and social affairs, which was recently emphasised in a discussion paper of the Association of German Cities 2023 [109], opens up the opportunity to combine heat prevention with health equity as a co-benefit.

**Info box 2 Environmental Justice Strategy of the state of Berlin**

In 2008, a model project on environmental justice in the state of Berlin was launched with the aim of creating a basis for policy decisions related to the social area. The environmental justice strategy consists of three action levels: (1) monitoring, (2) planning, and (3) implementation. It aims to analyse the socio-spatial distribution of environmental burdens and resources and to reduce or avoid burdens through compensatory concepts. At the small-scale neighbourhood level, the monitoring system tracks three indicators of factors detrimental to health (noise, air pollution, heat), one indicator for a health-promoting resource (availability of green spaces), and one social index indicator to determine social disadvantage, calculated from indicators on unemployment and transfer payments (for the employed and children under 15). In the Berlin Environmental Justice Atlas, these indicators are spatially mapped and intersections visualised, showing those urban areas that have particular or multiple burdens. The data of the Environmental Justice Atlas was last updated in 2021/2022 [70]. The Berlin Senate and the Berlin districts use the findings of the environmental justice monitoring to plan and implement measures in various areas (mobility, urban green spaces, construction, or housing). These measures are intended to contribute to increasing the quality of life and reducing environmental burdens. The Environmental Justice Strategy of the state of Berlin thus creates a starting point to further advance socially just, health-promoting, and environmentally friendly urban planning, based on real data. In the district of Friedrichshain-Kreuzberg, for example, the Environmental Justice Atlas was used to prioritise applications for residential areas without through traffic. In the current coalition agreement, the Berlin Senate refers to its intention to reduce the number of areas with multiple burdens by the end of the administrative period 2021 to 2026.

In future, the environmental justice strategy could benefit from integrating further environmental justice indicators, such as procedural, participatory, or recognition justice, into the monitoring system. Further dimensions could also be included, such as the proportion of very old persons and those with chronic pre-existing diseases or the density of social facilities such as daycare centres.

‣ Website Environmental Justice of the Berlin Senate Department for Urban Mobility, Transport, Climate Action, and the Environment

## Key statement

- Environmental and climate issues cannot be seen in isolation from social justice issues.
- Social inequalities in exposure to climate change impacts, biological sensitivity, and adaptive capacities have a combined effect on health.
- Anchoring climate justice in climate mitigation and adaptation requires an intersectionality perspective.
- A transformation to resilient, climate-just, sustainable, and health-promoting living environments for everyone is needed now.

## Figures and Tables

**Table 1:** Social differences in exposure according to articles of the German status report on climate change and health Source: Own representation

	Social determinants according to PROGRESS-Plus							Pregnant women and the unborn	People with preexisting diseases	Lifestyle factors
	Residence	Occupation	Gender	Social capital	Socioeconomic status	Age	People with disabilities			
**Impact of climate change on communicable diseases through…**										
Vector-borne diseases [56]	**↑** Certain vector-borne diseases occur in risk areas (GE, EE)	**↑** For professionals working in nature, e.g. in the forest, for employees in pest control companies, and others	**↑** Hantaviruses and tick-borne diseases in men (GE)			**↑** Hantaviruses in adults aged between 20 and 60 (GE)				**↑** Among people who spend a lot of time in nature, e.g. on walks (GE), mushroom picking, members of Scouting associations, geocachers
Waterborne infections and intoxications [57]						**↑** Cyanobacteria in children				**↑** During leisure activities, e.g. water sports
Foodborne infections and intoxications [58]										**↑** Campylobacter when barbecuing and bathing in surface waters (IE) **↑** Salmonella at barbecues and picnics
**Impact of climate change on non-communicable diseases through…**										
Temperature changes (heat) [60]	**↑** In urban areas, e.g. due to heat island effects (GE)	**↑** For people working outdoors, e.g. in agriculture or in the construction sector (EE) **↑** For health workers (EE, IE-R)			**↑** Due to unfavourable housing situations or homelessness (EE)					**↑** For people engaging in outdoor sports (EE)
Extreme weather events [61]	**↑** Risk areas for certain types of extreme weather events (e.g. storm surges near the coast)	**↑** Hazards for emergency services (IE-R) **↑** Among people working in agriculture during droughts (IE-R)	**↑** Dangers for women from loss of public order (IE-R)		**↑** Among people with low socioeconomic status	**↑** Dangers for children and older persons from loss of public order (IE-R)			**↑** Hazards due to inaccessibility of health facilities (IE-R)	
UV radiation [62]		**↑** For employees with external activities (GE)								**↑** For people who spend a lot of time outdoors (GE)
Allergen exposure [63]		**↑** In jobs in forestry and landscape management (GE) **↑** To mould allergens in workers carrying out renovation work after flood events (IE)								
Air pollutant exposure [64]	**↑** To PM and NO_x_ in urban agglomerations and at highly congested locations (GE) **↑** To ozone in suburban and rural areas (GE)									

**↑**=higher exposure, EE=European evidence, GE=German evidence, IE=international evidence, -R=review article, NOx=nitrogen oxides, PM=particulate matter

Only those PROGRESS-Plus dimensions are listed in the columns for which there was at least one statement on differences in exposure, sensitivity, or adaptive capacity. In the article on antimicrobial resistance, no statements were made regarding social differences in exposure.

**Table 2:** Social differences in sensitivity according to articles of the German status report on climate change and health Source: Own representation

	Social determinants according to PROGRESS-Plus							Pregnant women and the unborn	People with preexisting diseases	Lifestyle factors
	Residence	Occupation	Gender	Social capital	Socioeconomic status	Age	People with disabilities			
**Impact of climate change on communicable diseases through…**										
Vector-borne diseases [56]						**↑** In older persons for a neuroinvasive form of disease following West Nile virus infection (IE-R) **↑** In adults for severe tick-borne encephalitis after tick bites		**↑** Malformations in the foetus in Zika virus infection	**↑** In people with pre-existing diseases for a neuroinvasive form of disease after West Nile virus infection (IE-R)	
Waterborne infections and intoxications [57]			**↑** In older persons, especially males, for pneumonia caused by *Legionella* (Legionnaires’ disease)			**↑** In older persons for severe wound and soft tissue infection and sepsis following infection with non-cholera *Vibrio* (GE) **↑** In older persons for pneumonia caused by *Legionella* (Legionnaires’ disease)			**↑** In people with pre-existing diseases for severe wound and soft tissue infection and sepsis following infection with non-cholera *Vibrio* (GE) **↑** In people with pre-existing diseases for pneumonia caused by *Legionella* (Legionnaires’ disease)	**↑** In smokers for pneumonia caused by *Legionella* (Legionnaires’ disease)
Foodborne infections and intoxications [58]						**↑** In young and older persons after ingestion of pathogenic vibrions **↑** In infants, young children and the older persons after ingestion of parasites (IE-R)		**↑** In pregnant women after ingestion of pathogenic vibrions	**↑** In people with pre-existing diseases after ingestion of pathogenic vibrions **↑** In immunocompromised persons after ingestion of parasites (IE-R)	
**Impact of climate change on non-communicable diseases through…**										
Temperature changes (heat) [60]			**↑** Different effects of heat on the cardiovascular system in women and men with pre-existing diseases (GE)			**↑** In older persons (>65 years EE; >75 years GE), infants and young children (EE) **↑** In children and adolescents for psychological effects of heat (IE-R)		**↑** In pregnant women during heatwaves, as these can lead to premature births and low birth weight (EE, IE-R)	**↑** In people with pre-existing diseases, especially cardiovascular disease, respiratory disease, kidney disease, obesity, diabetes (GE, EE, IE-R) **↑** In people with pre-existing mental illness for psychological effects of heat (IE-R)	
Extreme weather events [61]			**↑** In female subjects for psychological effects of extreme weather events (IE-R)	**↑** In persons with intra-family conflicts, with little social support, with loss of social network for psychological effects of extreme weather events (IE-R)	**↑** In persons with low socioeconomic status for psychological effects of extreme weather events (IE-R)	**↑** In children and adolescents for psychological effects of extreme weather events (IE)	**↑** In people with physical limitations due to occurring physical burdens	**↑** In pregnant women due to stressful experiences, as these can lead to postnatal complications and long-term observable developmental delays in the children (IE-R)	**↑** In persons with pre-existing mental illness for psychological effects of extreme weather events (IE)	
UV radiation [62]						**↑** In children for damage to the eyes and skin (GE)				
Allergen exposure [63]									**↑** In people with pre-existing allergic rhinoconjunctivitis (high risk of developing allergic asthma) (IE)	
Air pollutant exposure [64]					**↑** In persons with low social status (IE-R)	**↑** In infants, children, and older persons (IE-R)		**↑** In pregnant women, i.e. an increased risk of pre-term birth and low birth weight (IE-R)	**↑** In people with chronic pre-existing diseases, especially chronic respiratory diseases and cardiovascular diseases (IE-R)	**↑** Smoking status and other lifestyle factors (IE-R)

**↑**=increased sensitivity, EE=European evidence, GE=German evidence, IE=international evidence, -R=review article

Only those PROGRESS-Plus dimensions are listed in the columns for which there was at least one statement on differences in exposure, sensitivity, or adaptive capacity. In the article on antimicrobial resistance, no statements were made regarding social differences in sensitivity.

**Table 3:** Social differences in adaptive capacity according to articles of the German status report on climate change and health Source: Own representation

	Social determinants according to PROGRESS-Plus							Pregnant women and the unborn	People with preexisting diseases	Lifestyle factors
	Residence	Occupation	Gender	Social capital	Socioeconomic status	Age	People with disabilities			
**Impact of climate change on non-communicable diseases through…**										
Temperature changes (heat) [60]	**↓** For people living in areas with high levels of soil sealing	**↓** For people working outdoors		**↓** For people in social isolation, especially in old age (EE)	**↓** For people with low socioeconomic status (EE)	**↓** For people in social isolation, especially in old age (EE)	**↓** For people with impairments (physical or mental) or with functional limitations (bedridden or placed in care) (EE)			
Extreme weather events [61]			**↓** In men, as they show a higher risk tolerance during events (GE) **↓** In women, as the long-term recovery is lower (GE)	**↓** Social networks increase resilience and civil emergency aid in case of disaster (IE)	**↓** For people with low socioeconomic status **↓** For people with lower financial status, as the long-term recovery is lower (GE)	**↓** For older persons and children, as they may not be able to get to safety independently (GE)	**↓** For people with physical or mental disabilities, as they may not be able to get to safety independently (GE)		**↓** For people with pre-existing diseases, as health system facilities may be inaccessible (IE-R)	

**↓**=lower adaptive capacity, ↑=higher adaptive capacity, EE=European evidence, GE=German evidence, IE=international evidence, -R=review article

Only those PROGRESS-Plus dimensions are listed in the columns for which there was at least one statement on differences in exposure, sensitivity, or adaptive capacity. The articles on the effects of climate change on communicable diseases and on effects on non-communicable diseases due to UV radiation, allergen exposure, and air pollution did not contain any statements regarding adaptive capacity.

**Table 4:** Recommendations for action and research needs for more climate justice Source: Own representation

Recommendation	Explanation
Systematically consider climate justice and anchor it as a cross-cutting issue in climate protection and climate adaptation	‣ Systematically consider social justice and health equity in the design and implementation of climate change mitigation and adaptation measures and policy programmes from an intersectionality perspective [17, 80] ‣ Use intervention-oriented guiding principles from environmental justice [24] and gender justice [74] as starting points In 2017, the Sixth Ministerial Conference on Environment and Health already called for equity to be considered as a cross-cutting dimension in all measures [99]. Municipal heat-health action plans have the potential to act as an instrument for climate justice (Info box 1).
Use established instruments for integrating climate and environmental justice into all spatially effective and socially relevant fields of action and for counteracting social segregation in municipalities	‣ Use informal spatial planning instruments in particular, such as integrated urban development concepts, to achieve more climate and environmental justice; instruments of e.g. housing policy can help minimise segregation effects ‣ At a strategic level, expand the Health in All Policies approach to become a societal responsibility for health, climate, and environmental justice [24]; practical guidelines for linking to established instruments can be found, for example, in the environmental justice toolbox [100]
Increase adaptive capacities and participation opportunities of socially disadvantaged population groups	‣ Enable socially disadvantaged population groups, who are often more frequently or severely affected by climate change impacts and may be more susceptible and have lower adaptation capacities, to participate in development and decision-making processes on climate adaptation strategies ‣ Support these populations in participation and in strengthening their adaptive capacities This includes, among others, target group-specific approaches to increasing participation opportunities and strengthen health literacy as well as measures for climate-just adaptation of working conditions, housing, cities, etc. for all people.
Counteract socially unequal biological sensitivity	‣ Address socially disadvantaged circumstances associated with multiple environmental burdens, psychosocial stress, lack of material resources and access to healthcare, which increase sensitivity to adverse climate change impacts, with policies for greater social justice, environmental justice, and healthcare for all
Establish integrated monitoring for decision support	‣ Develop, test, and consolidate approaches for integrated health, climate, environmental, and social monitoring at municipal, state, and federal level to support decision-making in interdepartmental cooperation in line with a Health in All Policies approach ‣ Enable the identification of (small-scale) multiple burdens, of social inequalities in adaptive capacity and in participation in decision-making processes through integrated monitoring ‣ Anchor an intersectionality perspective in integrated monitoring to capture the interaction of different inequalities This can build on a variety of activities in Germany, such as the Berlin Environmental Justice Atlas (Info box 2).
Systematically evaluate inequality effects	‣ Systematically evaluate the implementation and impact of climate change mitigation and adaptation measures from an equity perspective (equity impact assessment) and take immediate action in case of unintended negative effects on social inequalities and health equity [101, 102] Equity-focused health impact assessment tools could be useful [103]. Data from integrated health, climate, environmental, and social monitoring as mentioned above can be used.
Counteract stigmatisation of population groups	‣ In research and measures in the context of climate change and health, actively address possible stigmatisation of socially disadvantaged groups or people who have migrated due to climate change impacts
Develop and expand research approaches on mechanisms for the emergence of climate injustice and on the quantification of the impact of climate action on health equity	‣ Conduct research guided by interdisciplinarity and intersectionality [17, 74, 104] on processes of disadvantage and discrimination in exposure to climate change impacts and in climate mitigation and adaptation measures, in order to understand the effects of climate action on health equity (examples from research on heat-health action plans: [105, 106]), the mechanisms of emergence of climate injustice and of social destabilisation from climate change impacts [107]

**Annex Table 1:** Search query for the literature search on models/concepts in the area of climate change, health, and social justice in the database Medline via PubMed

Database	Search query
PubMed	“climate change”[Title/Abstract] AND health[Title/Abstract] AND (soci*[Title/Abstract] AND (justice[Title/Abstract] OR injustice[Title/Abstract] OR inequit*[Title/Abstract] OR equit* [Title/Abstract] OR inequalit*[Title/Abstract] OR equalit* [Title/Abstract])) AND (model [Title/Abstract] OR framework [Title/Abstract] OR concept* [Title/Abstract])

**Annex Table 2:** Search query for the systematic literature search on evidence in Germany in the Medline database via PubMed Keywords within each domain (social justice, climate change, Germany) with OR connection, between domains with AND

Area	Search query
Social justice	equit*[Title/Abstract] OR inequ*[Title/Abstract] OR “social advantage”[Title/Abstract] OR “social disadvantage”[Title/Abstract] OR “social exclusion”[Title/Abstract] OR “social inclusion”[Title/Abstract] OR “social status”[Title/Abstract] OR equal*[Title/Abstract] OR “social position”[Title/Abstract] OR “social gradient”[Title/Abstract] OR “social determinant” [Title/Abstract] OR social discrimination[MeSH Terms] OR deprivation[Title/Abstract] OR deprived[Title/Abstract] OR “socioeconomic advantage”[Title/Abstract] OR “socioeconomic disadvantage”[Title/Abstract] OR “socioeconomic exclusion”[Title/Abstract] OR “socioeconomic inclusion”[Title/Abstract] OR “socioeconomic status”[Title/Abstract] OR “socioeconomic position”[Title/Abstract] OR “socioeconomic gradient”[Title/Abstract] OR “socioeconomic determinant” [Title/Abstract] OR “socioeconomic discrimination”[Title/Abstract] OR “socio-economic advantage”[Title/Abstract] OR “socio-economic disadvantage”[Title/Abstract] OR “socio-economic exclusion”[Title/Abstract] OR “socio-economic inclusion”[Title/Abstract] OR “socio-economic status”[Title/Abstract] OR “socio-economic position”[Title/Abstract] OR “socio-economic gradient”[Title/Abstract] OR “socio-economic determinant”[Title/Abstract] OR “economic advantage”[Title/Abstract] OR “economic disadvantage”[Title/Abstract] OR “economic exclusion”[Title/Abstract] OR “economic inclusion” [Title/Abstract] OR “economic status”[Title/Abstract] OR “economic position”[Title/Abstract] OR “economic gradient” [Title/Abstract] OR “economic determinant”[Title/Abstract] OR “economic discrimination”[Title/Abstract] OR just*[Title/Abstract] OR injust*[Title/Abstract]
Climate change	climat*[Title/Abstract] OR climate change[MeSH Terms] OR “global warming”[Title/Abstract] OR extreme weather [MeSH Terms] OR “environmental change”[Title/Abstract] OR “ecological change”[Title/Abstract] OR greenhouse effect [MeSH Terms] OR cold[Title/Abstract] OR cool[Title/Abstract] OR cooling[Title/Abstract] OR heat[Title/Abstract] OR humid* [Title/Abstract] OR ice[Title/Abstract] OR temperature[Title/Abstract] OR “thermal comfort”[Title/Abstract] OR “thermal stress”[Title/Abstract] OR rain*[Title/Abstract] OR season* [Title/Abstract] OR snow*[Title/Abstract] OR “carbon emission” [Title/Abstract] OR warm[Title/Abstract] OR warming[Title/Abstract] OR wind[Title/Abstract] ultraviolet rays[MeSH Terms] OR clouds[Title/Abstract] OR flood*[Title/Abstract] OR drought[Title/Abstract] OR storm[Title/Abstract]
Germany	Germany[MeSH Terms] OR Germany[Title/Abstract] OR Germany[Other Term]
